# An Explanation

**Published:** 1854-04

**Authors:** 


### AN EXPLANATION.

Some of the proof-sheets of our last number were worked off without being submitted to our inspection, the consequence of which was an unusual number of typographical errors, and the omission of credits in our department of selections. We trust our cotemporaries will not doubt, that it is our earnest wish to do them entire justice, and that where there has been a failure to give the credits due it has arisen from the carelessness of the printer. We hope such omissions will not often occur.

